# Genome-wide association study of salt tolerance at the seed germination stage in rice

**DOI:** 10.1186/s12870-017-1044-0

**Published:** 2017-05-30

**Authors:** Yingyao Shi, Lingling Gao, Zhichao Wu, Xiaojing Zhang, Mingming Wang, Congshun Zhang, Fan Zhang, Yongli Zhou, Zhikang Li

**Affiliations:** 1 Institute of Crop Sciences/National Key Facility for Crop Gene Resources and Genetic Improvement, Chinese Academy of Agricultural Sciences, 12 South Zhong-Guan-Cun Street, Beijing, 100081 China; 2 Anhui Agricultural University, 130 West Chang-Jiang Street, Hefei, 230036 China; 3 Shenzhen Institute of Breeding and Innovation, Chinese Academy of Agricultural Sciences, 7 Peng-Fei Road, Da-Peng District, Shenzhen, 518120 China

**Keywords:** Rice, Salt tolerance, Germination, Genome-wide association study

## Abstract

**Background:**

Improving the salt tolerance of direct-seeding rice at the seed germination stage is a major breeding goal in many Asian rice-growing countries, where seedlings must often establish in soils with a high salt content. Thus, it is important to understand the genetic mechanisms of salt tolerance in rice and to screen for germplasm with salt tolerance at the seed germination stage. Here, we investigated seven seed germination-related traits under control and salt-stress conditions and conducted a genome-wide association study based on the re-sequencing of 478 diverse rice accessions.

**Results:**

The analysis used a mixed linear model and was based on 6,361,920 single nucleotide polymorphisms in 478 rice accessions grouped into whole, *indica*, and non-*indica* panels. Eleven loci containing 22 significant salt tolerance-associated single nucleotide polymorphisms were identified based on the stress-susceptibility indices (SSIs) of vigor index (VI) and mean germination time (MGT). From the SSI of VI, six major loci were identified, explaining 20.2% of the phenotypic variation. From the SSI of MGT, five major loci were detected, explaining 26.4% of the phenotypic variation. Of these, seven loci on chromosomes 1, 5, 6, 11, and 12 were close to six previously identified quantitative gene loci/genes related to tolerance to salinity or other abiotic stresses. The strongest association region for the SSI of MGT was identified in a ~ 13.3 kb interval (15450039–15,463,330) on chromosome 1, near salt-tolerance quantitative trait loci controlling the Na^+^: K^+^ ratio, total Na^+^ uptake, and total K^+^ concentration. The strongest association region for the SSI of VI was detected in a ~ 164.2 kb interval (526662–690,854) on chromosome 2 harboring two nitrate transporter family genes (*OsNRT2.1* and *OsNRT2.2*), which affect gene expression under salt stress. The haplotype analysis indicated that *OsNRT2.2* was associated with subpopulation differentiation and its minor/rare tolerant haplotype was detected.

**Conclusions:**

These results provide valuable information for salt tolerance-related gene cloning and for understanding the genetic mechanisms of salt tolerance at the seed germination stage. This information will be useful to improve the salt tolerance of direct-seeding rice varieties by genomic selection or marker-assisted selection.

**Electronic supplementary material:**

The online version of this article (doi:10.1186/s12870-017-1044-0) contains supplementary material, which is available to authorized users.

## Background

Rice (*Oryza sativa* L.), as the most important cereal crop, is a staple food for more than half the world’s population, especially in developing countries. Because labor costs and water crises have increased in recent years, there is a growing demand for low-input direct-seeded rice in many growing regions [1]. Soil salinization is a serious problem worldwide and a key abiotic stress in agriculture [2]. Salinity affects rice growth during all developmental stages from seed germination to reproduction [3]. With the development and spread of direct-seeding technology, which requires high levels of seedling establishment in rice paddy fields that are salinized to some degree, salt tolerance at the seed germination stage has become a major rice breeding goal in many Asian countries. Therefore, it is necessary to understand the genetic mechanisms of salt tolerance at the seed germination stage in rice.

Salt tolerance is a quantitative genetic characteristic that is controlled by multiple genes in rice [4]. In recent decades, with the rapid development of molecular marker technology, a number of quantitative trait loci (QTLs) for salt tolerance in rice have been identified using bi-parental linkage mapping of plants at different developmental growth stages, such as at the seedling and mature stages [5]. More than 70 QTLs controlling salt-related traits including the Na^+^/K^+^ ratio and survival time have been reported for rice [5]. Salt tolerance at the germination stage is not significantly correlated with salt tolerance at other stages [4]. To date, few studies have focused on evaluating the salt tolerance of rice at the germination stage to identify QTLs related to salt tolerance at this stage [6, 7]. A previous study detected only 16 QTLs associated with seed germination traits under salt stress and control conditions in a recombinant inbred rice population [7].

Linkage analysis of QTLs uses bi-parental genetic mapping populations. To date, most studies on QTLs associated with salt tolerance have examined individual mapping populations [5, 8]. However, this method is insufficient to reveal the genetic variation in salt tolerance among the rice germplasm. Over the past several years, genome-wide association studies (GWASs) using high-density genome-wide single nucleotide polymorphisms (SNPs) detected by next-generation sequencing have provided a powerful strategy to detect variants that can be used directly to improve rice varieties [9–12]. Recently, the 3000 rice genomes project (3 K RGP) [13] used Illumina next generation sequencing of a core collection of 3024 rice accessions from 89 countries to generate sequence data with high coverage (~94%) and mapping rate (~92.5%) and to construct a high-density SNP database [14] providing genotype data for GWAS of agronomic traits in rice. Several GWASs of salt tolerance in rice at the early tillering stage [15, 16] and reproductive stage [17] have been reported. However, no studies have evaluated the diverse rice germplasm to identify potentially novel loci for salt tolerance at the seed germination stage.

In this study, therefore, 478 rice accessions with an appropriate growth period and without distinct unfavorable traits in southern China selected from 3024 rice genomes sequenced by 3 K RGP [13], were used to conduct an association analysis of salt tolerance at the germination stage. For this analysis, we used 6,361,920 SNPs filtered from the 3 K RG 6.5mio SNP dataset in the Rice SNP-Seek Database [14]. The goal of this study was to understand the genetic basis and differentiation of salt tolerance at the germination stage in rice. This information will be useful for marker-assisted selection of direct-seeding rice varieties suitable for cultivation in salinized paddies.

## Results

### Seed germination of rice accessions under salt stress

The germination index (GI), vigor index (VI), germination rate (GR), mean germination time (MGT), and imbibition rate (IR) were determined for 35 rice accessions randomly selected from the whole panel under three salt treatments (60, 80 and 100 mM NaCl) (Additional file 1: Table S1). The GI, VI, and GR at 5 d (GR-5d) and GR at 10 d (GR-10d) significantly decreased as the NaCl concentration increased, and MGT was significantly higher in the 100 mM NaCl treatment than in the 60 and 80 mM NaCl treatments. These findings suggested that the seed germination capability was positively correlated with GI, VI, GR-5d, and GR-10d, and negatively correlated with MGT. The IR values at 24 h (IR-24 h) and 48 h (IR-48 h) were not significantly different among the three NaCl treatments. The seeds of 23 accessions germinated in the 60 mM NaCl treatment, but the seeds of only 11 and 3 accessions germinated in the 80 and 100 mM NaCl treatments, respectively (Additional file 2: Table S2). Thus, the 60 mM NaCl treatment was used in further experiments to compare germination traits among the 478 accessions (Additional file 2: Table S2).

The distributions of the values of seed germination-related traits under stress and control conditions, and the stress susceptibility index (SSI) to salt across the whole panel, are shown in Additional file 3: Figure S1. The SSI has been used to define the varieties or genotypes that show superior performance under stress and non-stress conditions in wheat [18] and rice [17, 19]. Lower SSI values indicate higher tolerance to stress. All the traits showed a continuous distribution, and many accessions were susceptible to salt stress. The traits could be roughly classified into two categories; one with a symmetrical distribution and one with a skewed distribution. Three traits (IR-24 h, IR-48 h, and GR-10d) under salt stress, one trait (VI) in the control, and one SSI trait (GR-10d) showed approximately symmetrical distributions. The other 16 traits (GI, VI, MGT, and GR-5d under salt stress; GI, MGT, IR-24 h, IR-48 h, GR-5d, and GR-10d in the control; and the SSIs of GI, VI, MGT, IR-24 h, IR-48 h, and GR-5d) showed highly or moderately skewed distributions (Additional file 3: Figure S1). Compared with their respective values in the control, the average values of GI, VI, GR-5d, and GR-10d under salt stress were decreased by 58.5%, 75.7%, 57.0% and 54.8%, respectively, while the MGT was increased by 14.6% across the whole population. However, the IR values at 24 h and 48 h under salt-stress conditions were roughly the same as that in the control.

The results of a correlation analysis among the seven seed germination traits across the whole panel are shown in Table 1. There were significant positive correlations between VI and GR-related traits (such as GI, GR-5d, and GR-10d) (Table 1), and the correlations were stronger (higher correlation coefficient values) under salt-stress conditions. There were significant negative correlations between MGT and the other traits in the control, and the correlation coefficient values were lower under salt-stress conditions. There was a strong correlation between IR-24 h and IR-48 h, but not between IR (IR-24 h and IR-48 h) and the other germination-related traits in both the control and the salt treatment. The correlation analysis indicated that all traits, except for IR, exhibited a salt-stress response in this study. Uncovering novel loci for these traits at the seed germination stage has great significance in rice breeding for salt tolerance.

**Table 1:** Correlation coefficients of paired traits evaluated under control and salt-stress conditions

	GI	VI	MGT	IR-24 h	IR-48 h	GR-5d	GR-10d
GI	0.198	**0.971**	−0.538	*−0.009*	*0.068*	**0.980**	**0.966**
VI	**0.823**	0.218	−0.494	*−0.002*	*0.088*	**0.959**	**0.948**
MGT	**-0.929**	**−0.756**	0.259	*−0.079*	*−0.118*	−0.537	-0.402
IR-24 h	*0.083*	*0.053*	*−0.123*	**0.836**	**0.870**	*0.006*	*-0.013*
IR-48 h	0.185	0.160	−0.213	**0.868**	**0.827**	*0.070*	*0.053*
GR-5d	**0.830**	0.686	**−0.831**	*0.073*	0.156	0.206	**0.960**
GR-10d	0.672	0.562	−0.413	*−0.025*	*0.044*	0.647	*0.121*

Upper and lower triangles indicate correlation coefficients under salt stress and control conditions, respectively. Diagonal values indicate correlation coefficients between control and salt stress conditions. All correlation coefficients are significant at *p* < 0.01 except for values in italics; values in bold indicate strong correlation (*r* > 0.8). GI, germination index; VI, vigor index; MGT, Mean germination time; IR, imbibition rate; GR, germination rate

**Fig. 1:**
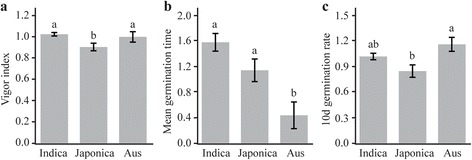
Comparison of stress susceptibility indices of seed germination traits with significant differences among accessions in different subgroups. Vigor index (**a**). Mean germination time (**b**). 10d germination rate (**c**). Figure shows average values ± SE. Multiple comparison tests were based on Duncan’s test at *P* < 0.01. Different letters above bars indicate significant differences among subgroups (*indica*, *japonica*, and *aus*)

There was highly significant genetic variation in all seed germination traits among rice germplasm accessions nested within the rice subgroups, together with less significant variance in GI, IR-24 h, IR-48 h, and GR-48d within subgroups (*indica*, *japonica*, *aus* and other) under salt stress (60 mM NaCl). There was no significant variance between replications for all seed germination traits (Table 2). The box plots of SSI indicated a substantial level of genotypic variation in salt-stress responses among different accessions (Additional file 4: Figure S2). An ANOVA demonstrated that the SSI of VI and GR at 10 d were significantly lower in the subgroup *japonica* than in the subgroups *indica* and *aus*, and the SSI of MGT was significantly lower in the *aus* subgroup than in the *indica* and *japonica* subgroups (Fig. 1). The average VI values for the *japonica*, *indica*, and *aus* subgroups were 6.48, 7.48, and 5.71, respectively, in the control, and 2.04, 1.68 and 1.35, respectively, under salt stress. This result suggested that the accessions in the *japonica* subgroup had higher salt tolerance than did the accessions in the other subgroups.

**Table 2:** ANOVA (*F*-values) of seven seed germination-related traits in 478 rice accessions under salt stress (60 mM NaCl)

Sources	Df	Germination index		Vigor index		Mean germination time		Imbibition rate				Germination rate			
								24 h		48 h		5d		10d	
Rep	1	0.6	ns	0.1	ns	2.6	ns	0.0	^b^	0.1	ns	0.7	ns	1.9	ns
Subgroup	3	3.3	^a^	2.2	ns	1.5	ns	4.0	^b^	6.0	^b^	2.6	ns	3.3	^a^
Accession(Subgroup)	474	436.4	^b^	485.7	^b^	385.2	^b^	392.0	^b^	390.9	^b^	707.5	^b^	571.3	^b^

^a^ and ^b^ indicate significance at 0.05 and 0.01 levels, respectively. Ns, not significant

### Loci associated with salt tolerance at the germination stage identified by GWAS

**Fig. 2:**
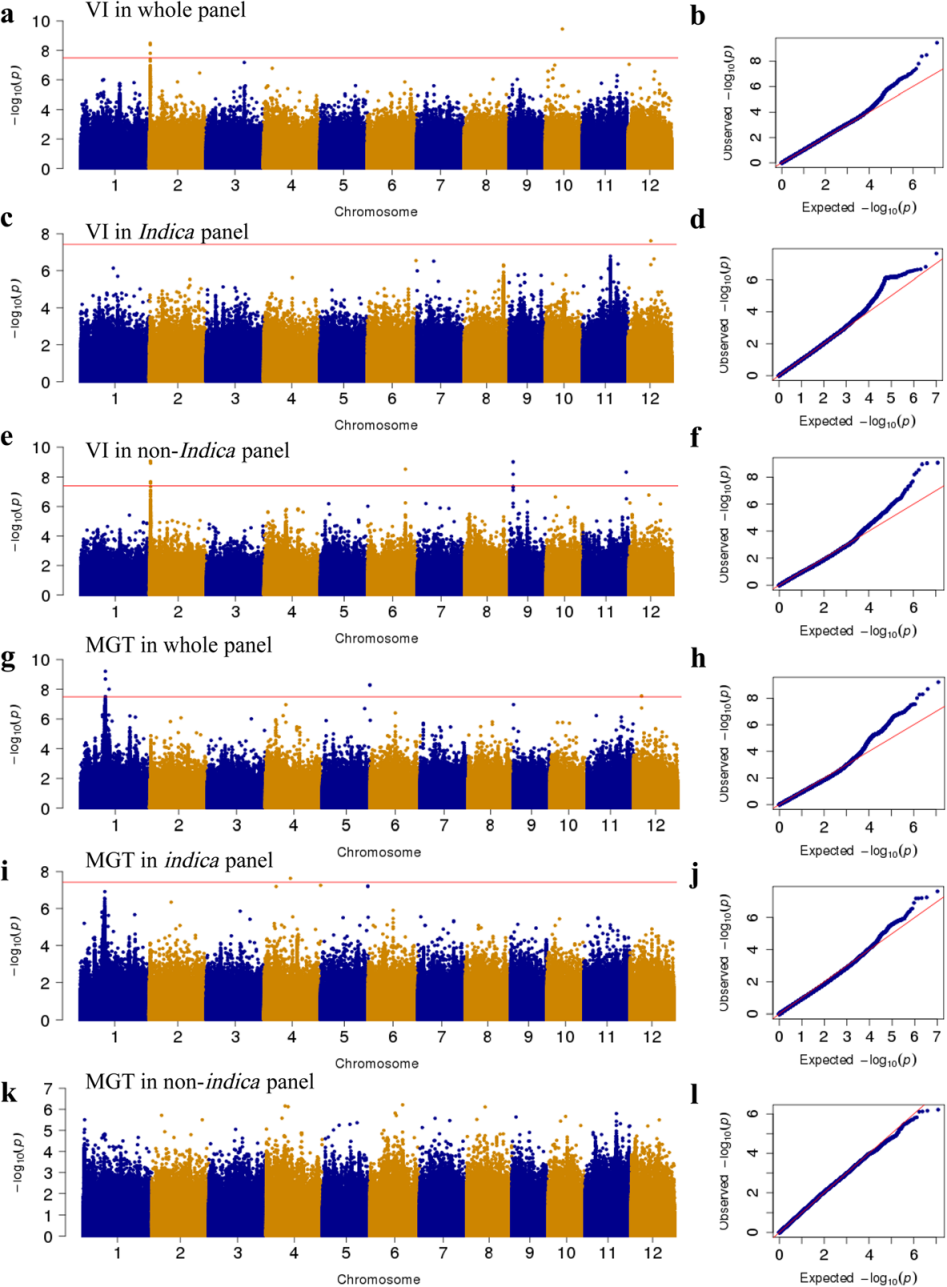
Genome-wide association mapping of rice salt tolerance at the germination stage. Manhattan and quantile–quantile plots for stress susceptibility indices (SSI) of vigor index in the whole (**a**, **b**), *indica* (**c**, **d**) and non-*indica* panels (**e**, **f**). Manhattan and quantile–quantile plots for SSIs of mean germination time in whole (**g**, **h**), *indica* (**i**, **j**) and non-*indica* panels (**k**, **l**). Red horizontal line indicates genome-wide significance threshold

We randomly selected 20% of SNPs with minor allele frequency (MAF) >10% and missing rate < 20% for the linkage disequilibrium (LD) decay analysis of each panel. The LD decayed quickly within ~8 kb for all panels, and decayed to its half-maximum within 95 kb for the whole panel, 52 kb for *indica*, and 230 kb for non-*indica* (Additional file 5: Figure S3). We selected 6,361,920 SNPs across the entire rice genome for the GWAS of seed germination traits under salt-stress and control conditions, and the related SSI. The LD blocks in all GWAS panels were defined by a fixed cutoff of <0.2 for the LD statistic *r* ^2^ on the same chromosome. Multiple significant SNPs in a LD block were clustered as one association locus in each panel, and the SNP with the minimum *P* value in a cluster was considered as the lead SNP. After Bonferroni multiple test correction, 11 loci (22 SNPs) were identified as being associated with the SSIs of the two seed germination traits (VI and MGT) at a significance level of 0.05 (Additional file 6: Table S3) in the whole, *indica*, and non-*indica* panels (Table 3 and Additional file 7: Table S4). The quantile–quantile plots for the GWAS results shown in Fig. 2 indicate that the model was well fitted to the data. Among the 11 loci, 7 were located in intervals containing previously mapped QTLs or genes associated with tolerance to salinity or other abiotic stresses (Table 3). Additionally, for GI, VI, MGT, GR-5d, and GR-10d in the control, 41, 136, and 7 significant SNPs were detected in the whole, *indica*, and non-*indica* panels, respectively. This result indicated that there is wide genetic variation in seed germination-related traits in the rice germplasm (Additional file 7: Table S4). Here, we focused on the significant associations of the SSIs of VI and MGT, the two parameters that underlie salt tolerance at the germination stage in rice.

**Table 3:** Summary of loci significantly associated with stress susceptibility indices of seed germination-related traits

Trait^a^	Chr.^b^	Number of significant signals^c^	Lead SNP position (bp)^d^	*P* value	Gene harbored lead SNP	Panel^e^	Allele^f^	Mean SSI		Known QTL/Trait
								Major	Minor	
MGT	1	3	15,450,039	6.22E-10	LOC_Os01g27710-LOC_Os01g27720	Whole	G/A	1.09	2.92	RM1-R886 [31]/Na^+^ uptake; K^+^ concentration; Na^+^: K^+^ ratio
MGT	1	1	17,736,489	9.93E-09	LOC_Os01g32330	Whole	C/T	1.23	2.57	
VI	2	7	666,962	1.15E-09	LOC_Os02g02190	Whole, non-*Indica*	A/G	0.98	0.31	
MGT	4	1	16,501,431	2.32E-08	LOC_Os04g27950-LOC_Os04g27960	*Indica*	C/T	1.57	1.47	
MGT	5	2	29,704,394	5.10E-09	LOC_Os05g51770	Whole	T/C	1.35	1.32	*qRGR-5* [43] /relative germination rate with ABA; *qGR5–1* [44] /Germination rate
VI	6	1	23,605,165	2.93E-09	LOC_Os06g39756-LOC_Os06g39750	non-*Indica*	G/A	1.08	0.27	RG716 [45]/Salinity tolerance
VI	9	2	2,214,950	9.71E-10	LOC_Os09g04210	non-*Indica*	A/T	0.94	0.52	
VI	10	1	10,723,028	3.54E-10	LOC_Os10g21100-LOC_Os10g21110	Whole	G/A	1.02	0.36	
VI	11	1	27,305,217	4.64E-09	LOC_Os11g45110-LOC_Os11g45120	non-*Indica*	T/C	1.01	0.37	RM206 [46]/Relative shoot elongation under submergence
MGT	12	2	5,000,802	2.88E-08	LOC_Os12g09510-LOC_Os12g09520	Whole	A/C	1.16	3.93	*qCTS12* [47]/Seedling cold tolerance; *rv12–2* [48]/Root volume at seedling stage
VI	12	1	14,215,505	2.41E-08	LOC_Os12g24780	*Indica*	C/T	1.06	0.92	*qtl12.1* [49] /Drought resistance

^a^ Traits for which significant signals were associated with stress susceptibility index of that trait. MGT, mean germination time; VI, vigor index

^b^ Chromosome number

^c^ Number of significant association signals detected in region

^d^ Representative SNP position on rice genome assembly MSU version 7.0

^e^ Panel containing significant GWAS sites

^f^ Major allele/minor allele

For the SSI of MGT, five significant associated loci were identified on chromosomes 1, 4, 5, and 12 in the whole, *indica*, and non-*indica* panels. These loci explained 26.4% of the phenotypic variation in the whole panel. Four loci on chromosomes 1, 5, and 12 were close to QTLs/genes identified previously as being related to tolerance to salinity or other abiotic stresses (Table 3 and Fig. 2). The genome-wide peak locus, which contained the lead SNP chr1_15,450,039 (*P* = 6.2 × 10^−10^) in the whole panel, was identified in a ~ 13.3 kb interval (15450039–15,463,330) on chromosome 1. This locus included two associated SNPs located in the intergenic region of *LOC_Os01g27710* (encoding an expressed protein) and *LOC_Os01g27720* (encoding an E3 ubiquitin-protein ligase, MGRN1) and one associated SNP in the coding sequence-missense variant of *LOC_Os01g27730* (encoding a GTPase with an unknown functional domain-containing protein) (Fig. 2 and Additional file 7: Table S4). The MGT of the minor genotype at the peak SNP chr1_15,450,039, AA (2.92 ± 2.87), was significantly greater than that of the major genotype, GG (1.09 ± 1.84). One significant locus on chromosome 4 containing SNP chr4_16,501,431 (*P* = 6.2 × 10^−8^) was detected only in the *indica* panel and was located in the intergenic region of *LOC_Os04g27950* (encoding an expressed protein) and *LOC_Os04g27960* (encoding a B3 DNA-binding domain-containing protein) (Additional file 7: Table S4).

**Fig. 3:**
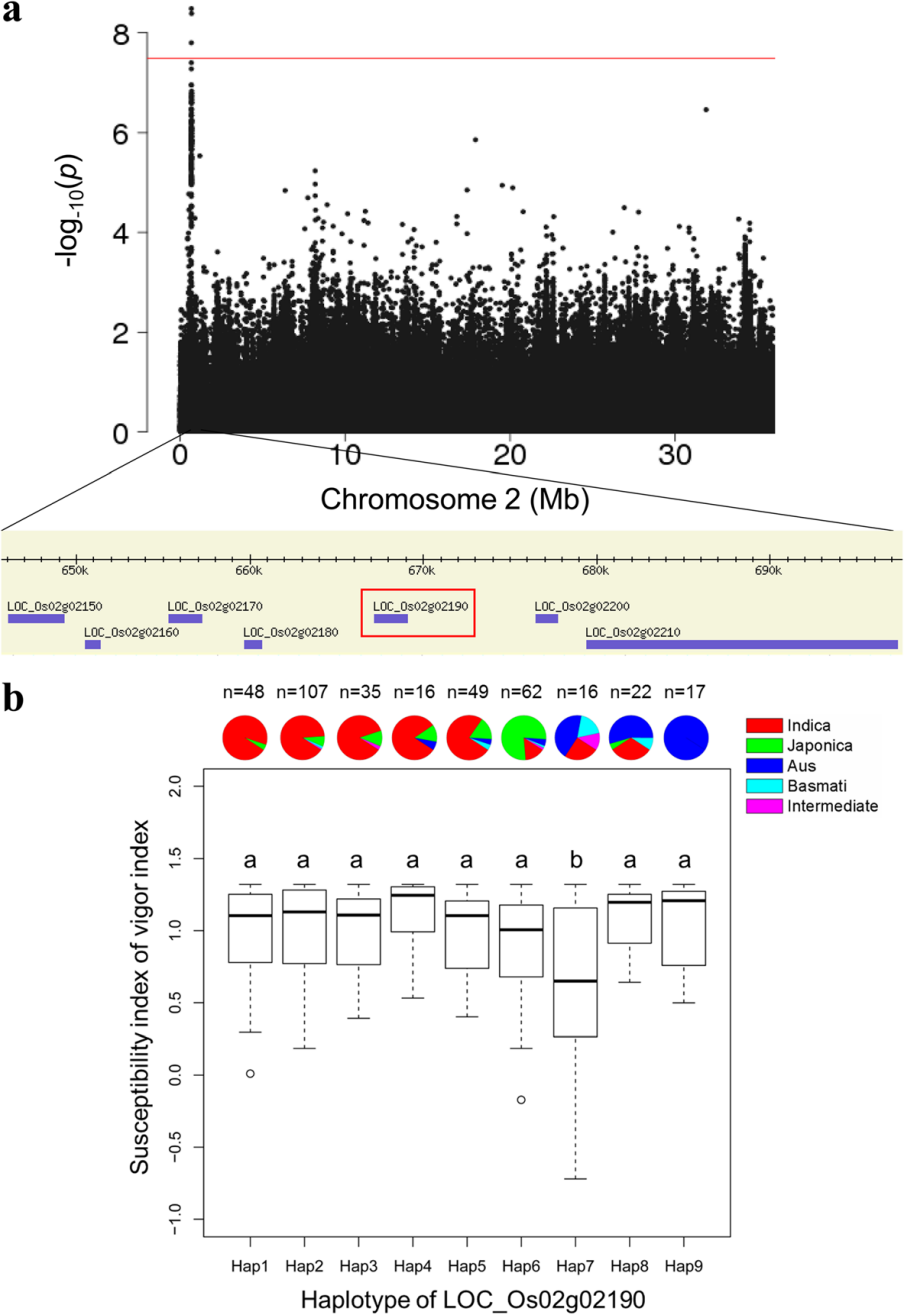
Strong association on rice chromosome 2 between stress susceptibility index and seed vigor index (**a**), and haplotype analysis of candidate gene *LOC_Os02g02190* (**b**)

For the SSI of VI, six significantly associated loci were identified on chromosomes 2, 6, 9, 10, 11, and 12 in the whole, *indica*, and non-*indica* panels. These loci explained 20.2% of the phenotypic variation in the whole panel. Three loci on chromosomes 6, 11, and 12 were close to previously identified QTLs related to tolerance to salinity or other abiotic stresses (Table 3 and Fig. 2). The strongest association in the hot region was observed on chromosome 2 in a ~ 164.2 kb interval (526662–690,854) that included three and four significant SNPs in the whole and non-*indica* panels, respectively (Additional file 7: Table S4). In this region, the lead SNP in the GWAS, chr2_666,962 (*P* = 1.2 × 10^−9^), was identified in the intergenic region of *LOC_Os02g02180* (retrotransposon protein) and *LOC_Os02g02190* (*OsNRT2.2*) (Fig. 3 and Additional file 7: Table S4). The SSI for the VI of the major susceptible genotype at the lead SNP (chr2_679199), AA (0.98 ± 0.32), was significantly greater than that of the minor resistant genotype, GG (0.31 ± 0.48), in the non-*indica* population. The haplotypes of *LOC_Os02g02190* (*OsNRT2.2*) were built based on 30 SNPs in the 2-kb upstream promoter region and 5′ untranslated region and synonymous SNPs in the coding region (Fig. 3 and Additional file 8: Table S5). In total, we detected nine haplotypes (Hap1–5 mainly in the *indica* panel, Hap6 mainly in the *japonica* panel, and Hap7–9 mainly in the *aus* panel) shared by more than 5 of the 372 accessions. Multiple comparison tests showed that accessions with Hap7 had lower SSIs of VI than did accessions with other haplotypes (Fig. 3). The 16 accessions with the tolerant haplotype (Hap7) of *OsNRT2.2* (*LOC_Os02g02190*) are mainly from Bangladesh and India and belong to the *aus*, *basmati*, *indica* and intermediate subgroups, but not the *japonica* subgroup. However, we did not find a significant difference in the SSIs of VIs among the accessions with different haplotypes of *LOC_Os02g02170* (*OsNRT2.1*), which also belongs to the NRT2 family and is close to *OsNRT2.2*.

Another significant peak region with the lead SNP chr9_2,214,950 (*P* = 9.7 × 10^−10^) in the non-*indica* panel was located in the intron region of *LOC_Os09g04210*, which encodes a zinc ion-binding protein (Table 3). The SSI of the VI of the major genotype at the lead SNP (chr9_2,214,950), AA (0.94 ± 0.38), was significantly greater than that of the major genotype, TT (0.52 ± 0.49).

## Discussion

### Variations in the salt tolerance of rice at the germination stage

Accurate phenotyping is the most important part of GWASs on rice [10]. Here, we measured seven seed germination-related traits (GI, VI, MGT, IR-24 h, IR-48 h, GR-5d, and GR-10d) under salt stress and control conditions. These traits have been used successfully in previous studies for the QTL mapping of salt tolerance at the rice germination stage [6, 7]. The intensity of the salt treatment is an important factor in the effective evaluation of salt tolerance during rice germination. We compared seven seed germination-related traits for 35 randomly selected rice accessions under three different NaCl concentrations (60, 80, and 100 mM NaCl) (Additional file 1: Table S1) and found that 60 mM NaCl was the appropriate treatment to detect sufficient levels of genetic variation among the 478 rice accessions used in this study. These accessions showed continuous segregation of seed germination-related trait distribution frequencies (Additional file 3: Figure S1).

Mondal and Pramanik evaluated the response of 35 rice varieties to salt stress during seed germination, and found that *japonica* varieties had higher tolerance than *indica* varieties to high-salt conditions [20]. Our results indicated that the SSIs of VI and GR at 10d were significantly lower in the *japonica* subgroup than in the *indica* and *aus* subgroups (Fig. 1), indicating that *japonica* varieties had higher tolerance to salt stress than did the other subgroups at the germination stage. Different genetic and physiological mechanisms of salt tolerance exist in the diverse germplasm of rice, and salt tolerance mechanisms can vary among varieties even within a particular subgroup [21, 22]. It is generally thought that higher uptake of K^+^ in *indica* varieties results their higher tolerance to salinity than *japonica* rice at the seedling stage [23, 24]. However, the mechanism underlying salt tolerance at the seed germination stage in rice is still obscure. Because seed germination is a complex trait [25], and, like salt tolerance, governed by multiple genes [5], it is difficult to improve seed germination under salt stress. Thus, to improve salt tolerance at the germination stage in rice, breeders should pyramid the tolerant SNP genotype at the loci associated with salt tolerance using the potentially tolerant varieties identified in this study. For successful, knowledge-based crop improvement, further studies should focus on understanding the correlation between salt tolerance at the seed germination stage and that at the seedling stage, and on elucidating the molecular mechanisms underlying salt tolerance at different growth stages in rice.

### QTLs and candidate genes associated with salt tolerance of rice at the germination stage

Linkage mapping using a bi-parental population benefits from high statistical power resulting from many individuals sharing the identical genotype at a given locus, but it has low resolution because of the limited number of recombination events. In contrast, association mapping has higher resolution because of the long recombination histories of natural populations, but has lower statistical power because most genotypes occur in only a few individuals [26]. The distance of LD is an important factor in determining the efficiency of GWAS [27]. In this study, more rapid LD decay was observed in the *indica* subgroup than in the non-*indica* subgroup comprising mainly *japonica* and *aus* (Additional file 5: Figure S3). This is consistent with the LD patterns reported for different subpopulations in rice [28, 29], suggesting that there has been a stronger bottleneck in *japonica* than in *indica* during domestication. The extent of LD decay detected in *indica* in this study (~50 kb) was smaller than that detected in another set of *indica* germplasm in another study (~100 kb) [29], partly because of the larger number of SNPs, which provides high resolution for GWAS.

To date, more than 70 QTLs related to salt tolerance in rice have been mapped using bi-parental linkage mapping populations [5], but this is the first study to use association mapping to detect potentially novel QTLs for salt tolerance at the seed germination stage. We identified 11 loci associated with salt tolerance at the germination stage in rice using a conservative threshold based on a Bonferroni-adjusted *P* value (0.05/total markers) to reduce false positives. We compared the loci identified in this study with previously reported QTLs related to salinity or other stresses in the QTL Annotation Rice Online (Q-TARO) database [30]. This comparison showed that seven loci on chromosome 1, 5, 6, 11, and 12 were co-located with six previously detected QTLs related to tolerance to salinity or other abiotic stresses in rice (Table 3). For example, Koyama et al. [31] reported an initial area, from markers RM1 to R886 on chromosome 1, which was associated with QTLs controlling the Na^+^: K^+^ ratio, total Na^+^ uptake, and total K^+^ concentration. Two regions significantly associated with the SSI of MGT on chromosome 1 were detected in this area in this study.

Our results demonstrate that the gene *OsNRT2.2* (*LOC_Os02g02190*), encoding a nitrate transporter, is a candidate gene in the hot region for the SSI of VI (on rice chromosome 2) in different GWAS panels. In rice, OsNAR2.1 interacts with OsNRT2.2 and plays a key role in enabling plants to cope with a variable nitrate supply [32]. Salt stress affects the expression level of the *OsNRT* gene family, which might contribute to the accumulation of NO_3_^−^ in the leaves of salt-stressed rice [33]. From the haplotype analysis, we found that *OsNRT2.2* (*LOC_Os02g02190*) was associated with subpopulation differentiation (Fig. 3). The minor/rare tolerant haplotype (Hap7) of *OsNRT2.2* (*LOC_Os02g02190*) could be useful in further studies and in breeding for salt tolerance at the rice germination stage.

## Conclusions

We conducted genome-wide association mapping for salt tolerance at the germination stage based on high-density SNPs using 478 rice accessions. We identified 11 loci using the SSIs of germination-related traits, which co-located with six previously detected rice QTLs related to tolerance to salinity or other abiotic stresses. The minor/rare salt-tolerant haplotype of *OsNRT2.2* (*LOC_Os02g02190*) was detected in a haplotype analysis. This gene is a candidate for the associated hot region on rice chromosome 2. Our study provides new insights into the genetic basis of salt tolerance in rice. Identification of varieties with high salt tolerance at the germination stage, as well as knowledge of the associated SNPs and haplotype, could be useful for rice production and for improvement of direct-seeding varieties.

## Methods

### Rice germplasms and evaluation of seed GRs under salt stress

We selected 478 accessions from 46 countries and areas with an appropriate growth period and without distinct unfavorable traits in southern China (Additional file 2: Table S2) in the 3 K RGP [13] to evaluate salt tolerance at the seed germination stage. In total, there were 305 *indica* accessions, 85 *japonica* accessions (9 *japonica*, 34 temperate *japonica*, and 43 tropical *japonica*), 65 *aus* accessions, 16 basmati accessions, and 7 intermediate accessions. All seeds used in your study were from the International Rice Genebank Collection at the International Rice Research Institute.

A total of 120 yellow-ripe seeds of each accession were dried at 50 °C for 3 days to break seed dormancy. The seeds were surface-sterilized with 15% sodium hypochlorite solution for 20 min and then rinsed three times with sterile distilled water before the germination experiment. Two replications for each treatment, each consisting of 30 seeds from each accession, were placed in 9-cm-diameter Petri dishes on two layers of filter paper, to which 10 mL of NaCl solution was added to simulate salt-stress conditions. In the control, the filter paper was soaked with 10 mL water. The seeds were incubated in a growth chamber at 30 °C under a 12-h light/12-h dark photoperiod with 80% relative humidity for 10 days. To determine the most suitable salt concentration, we selected 35 accessions for treatment with NaCl at three different concentrations (60, 80, and 100 mM). More genetic variation was observed in the 60 mM NaCl treatment than in the other treatments. Therefore, the seed GRs were evaluated among all the accessions under a 60 mM NaCl treatment and control (water) conditions. The solution was replaced every day to maintain the NaCl concentration and the distilled water volume.

Dry seeds, and seeds incubated for 24 and 48 h, were weighed independently to calculate seed IR (mg/g) using the method of Wang et al. [6], as follows: IR = (*W*_2_ – *W*_1_)/*W*_1_ × 1000, where *W*_1_ (g) represents the dry seed weight, and *W*_2_ (g) represents the total seed weight after imbibition for 24 h or 48 h. The seeds that germinated were observed each day to calculate the GR and GI. We considered a seed germinated when its shoot length was greater than half of the seed length and the root length was greater than the seed length. The GR was the germination percentage at a certain day (GR = *N*_t_/*N*_0_ × 100%, where *N*_t_ represents the number of germinated seeds at day *t* and *N*_0_ represents the total number of experimental seeds). The GI was calculated by the method of Wang et al. [6] as follows: GI = ∑(*G*_t_/*T*_t_), where *G*_t_ is the accumulated number of germinated seeds at day *t* and *T*_t_ is the time corresponding to *G*_t_ in days. The MGT was calculated for the GR using the following formula: MGT = ∑*T*_i_ *N*_i_/∑*N*_i_, where *N*_i_ is the number of newly germinated seeds at day *t* [34]. We chose 10 germinated seeds to measure the average shoot length after 10 days, and the seed VI was calculated using the formula [6]: VI = GI × average shoot length. The responses of the genotypes to salt stress were expressed according to the SSIs calculated using the methods of Fischer and Maurer [35] as follows: SSI = (1 − *Y*s/*Y*p)/D, where *Y*s = mean performance of a genotype under stress; *Y*p = mean performance of the same genotype without stress; D (stress intensity) = 1 − (mean *Y*s of all of the genotypes/mean *Y*p of all of the genotypes).

### LD decay

The 3 K RG 6.5mio SNP dataset was downloaded from the Rice SNP-Seek Database (http://www.oryzasnp.org/) [14]. We randomly selected 20% of SNPs with MAF > 10% and missing rate < 20% for the LD decay analysis of each panel (whole panel: 478 accessions with 683,037 SNP, *indica* panel: 305 accessions with 473,146 SNPs, and non-*indica* panel: 173 accessions with 720,819 SNPs). We calculated *r* ^2^ as an estimation of LD using plink software [36] with the parameter ‘--r2 --ld-window-kb 1000 --ld-window 99999 --ld-window-r2 0’, and the parameter ‘--thin 0.2’ to randomly select 20% of the SNPs.

### Genome-wide association mapping

A total of 6,361,920, 4,736,461, and 6,029,780 SNPs with a minor allele frequency > 5% and accession numbers with minor alleles ≥6 were filtered for the association analyses in the whole population, *indica*, and non-*indica* panels, respectively. The GWAS was performed with a linear mixed-effects model to determine the association between each SNP and the evaluated phenotypes and SSIs using an efficient mixed-model analysis with EMMA eXpedited (EMMAX) software [37]. An identical-by-state matrix based on genome-wide SNP data was used to create the kinship matrix that measured the genetic similarities between individuals. The effective number of independent markers (*N*) was calculated using GEC software [38], and significant thresholds (0.05/*N*) were calculated. The effective number of independent SNPs was 1,546,501, 1,336,375, and 1,255,892 in the whole, *indica*, and non-*indica* panels, respectively. Based on a significance level of 0.05, the genome-wide significance thresholds were *P* = 3.23 × 10^−8^, 3.74 × 10^−8^, and 3.98 × 10^−8^ for the whole, *indica*, and non-*indica* panels, respectively. To obtain independent association signals, multiple SNPs passing the threshold on the same chromosome were clustered as one association locus by *r* ^2^ of LD ≥ 0.2 in each panel, and the SNP with the minimum *P* value in a cluster was considered as the lead SNP. Candidate genes near hits were extracted from the literature and the Q-TARO database [30]. The phenotypic variation explained (*R* ^2^) by the multiple lead SNPs of association loci in the whole, *indica*, and non-*indica* panels was estimated by a linear regression using SAS PROC GLM [39] after coding one allele as 1 and the other as 0. Manhattan and Q–Q plots were created by the R package ‘qqman’ [40] using the GWAS results.

### Significant signal analyses and annotations

Synonymous and nonsynonymous SNPs, and SNPs with large effect changes, were annotated based on gene models of the IRGSP 1.0 ‘Nipponbare’ annotated reference genome [41] using snpEff software version 4.0 [42]. All significant SNPs located in genes and the annotation information from the IRGSP 1.0 ‘Nipponbare’ annotated reference genome [41] are listed in Additional file 8 and Table S5.

### Statistical analyses

Correlation analyses were conducted between pairs of tested traits using R software. Differences in phenotypic and SSI values of accessions among haplotypes or subgroups were evaluated by one-way ANOVA and Duncan’s multiple mean comparison test at the 5% level of significance using SAS [39].

## Additional files

Additional file 1: Table S1. Phenotypic evaluation of seed germination for 35 randomly selected rice accessions under different NaCl concentrations and in the control. (DOCX 15 kb)

Additional file 2: Table S2. Rice accessions used in this study and related phenotypic data. (XLSX 141 kb)

Additional file 3: Figure S1. Frequency distributions of germination index, vigor index, mean germination time, imbibition rate, and germination rate for 478 rice accessions under salt-stress and control conditions, and stress susceptibility indices of these traits. Under salt stress (a) and control (b) conditions. Stress susceptibility index (c). (TIFF 1093 kb)

Additional file 4: Figure S2. Box plots for stress susceptibility indices of germination-related traits in 478 rice accessions. (TIFF 1970 kb)

Additional file 5: Figure S3. Differences in linkage disequilibrium among panels. (TIFF 422 kb)

Additional file 6: Table S3. Filtered and effective numbers of SNPs across subpopulations and adjusted significant *P* value thresholds based on Bonferroni correction. (XLSX 9 kb)

Additional file 7: Table S4. List of SNPs with significant *P* values detected by genome-wide association analysis in this study. (XLSX 24 kb)

Additional file 8: Table S5. Haplotype analysis of *LOC_Os02g02190* using 31 SNPs in 2-kb upstream promoter region, 5′ and 3′ untranslated regions, and coding region based on IRGSP 1.0 ‘Nipponbare’ annotated reference genome. (XLSX 15 kb)

## Abbreviations

3 K RGP: 3000 rice genomes project
Chr: Chromosome
GI: Germination index
GR: Germination rate
GR-10d: Germination rate at 10 d
GR-5d: Germination rate at 5 d
GWAS: Genome-wide association study
Hap: Haplotype
IR: Imbibition rate
IR-24 h: Imbibition rate at 24 h
IR-48 h: Imbibition rate at 48 h
LD: Linkage disequilibrium
MAF: Minor allele frequency
MGT: Mean germination time
Q-TARO: QTL Annotation Rice Online
QTL: Quantitative trait loci
SNP: Single nucleotide polymorphism
SSI: Stress-susceptibility index
VI: Vigor index
