# Supplementary material for: Genome-wide association study of salt tolerance at the seed germination stage in rice
Source: BMC Plant Biol. 2017 May 30;17:92. doi: 10.1186/s12870-017-1044-0 (PMC5450148; doi:10.1186/s12870-017-1044-0)
Supplement: Supplementary file 1 — Phenotypic evaluation of seed germination for 35 randomly selected rice accessions under different NaCl concentrations and in the control. (DOCX 15 kb) [file 12870_2017_1044_MOESM1_ESM.docx]

**Table S1. Phenotypic evaluation of seed germination for 35 randomly selected rice accessions under different NaCl concentrations and in the control**

| NaCl concentration (mM) | Germination index | | |  | Vigor index | | |  | Mean germination time | | |  | Imbibition rate | | | | | | |  | Germination rate | | | | | | |
| --- | --- | --- | --- | --- | --- | --- | --- | --- | --- | --- | --- | --- | --- | --- | --- | --- | --- | --- | --- | --- | --- | --- | --- | --- | --- | --- | --- |
|  |  |  |  |  |  |  |  |  |  |  |  |  | 24h | | |  | 48h | | |  | 5d | | |  | 10d | | |
|  | Mean^a^ | SE | Range |  | Mean | SE | Range |  | Mean | SE | Range |  | Mean | SE | Range |  | Mean | SE | Range |  | Mean | SE | Range |  | Mean | SE | Range |
| Control | 0.94^a^ | 0.05 | 0.39~1.43 |  | 4.81^a^ | 0.30 | 1.80~9.17 |  | 4.25^b^ | 0.13 | 2.89~5.42 |  | 592^a^ | 25 | 350~1000 |  | 666^a^ | 25 | 400~1140 |  | 75.3^a^ | 3.8 | 30~100 |  | 85.4^a^ | 2.8 | 45~100 |
| 60 | 0.19^b^ | 0.04 | 0.00~1.07 |  | 0.50^b^ | 0.11 | 0.00~3.00 |  | 4.73^ab^ | 0.21 | 3.13~9.00 |  | 568^a^ | 13 | 330~690 |  | 601^b^ | 15 | 430~750 |  | 16.1^b^ | 3.6 | 0~93 |  | 17.9^b^ | 3.6 | 0~93 |
| 80 | 0.13^bc^ | 0.08 | 0.00~2.68 |  | 0.30^b^ | 0.17 | 0.00~5.88 |  | 4.99^ab^ | 0.13 | 4.29~7.00 |  | 596^a^ | 11 | 472~750 |  | 604^b^ | 14 | 412~780 |  | 8.0^bc^ | 2.9 | 0~80 |  | 8.6^c^ | 3.0 | 0~80 |
| 100 | 0.02^c^ | 0.01 | 0.00~0.40 |  | 0.03^b^ | 0.02 | 0.00~0.67 |  | 5.56^a^ | 0.09 | 5.00~6.00 |  | 556^a^ | 20 | 197~872 |  | 607^b^ | 19 | 197~830 |  | 1.4^c^ | 1.1 | 0~35 |  | 2.1^c^ | 1.4 | 0~45 |

^a^ Letters after the mean value of each trait are based on Duncan’s multiple range tests after one-way ANOVA. Different letters are statistically significant at the *P* < 0.05 level.
