# Supplementary figures and images for: Genome-wide association study of salt tolerance at the seed germination stage in rice
Source: BMC Plant Biol. 2017 May 30;17:92. doi: 10.1186/s12870-017-1044-0 (PMC5450148; doi:10.1186/s12870-017-1044-0)

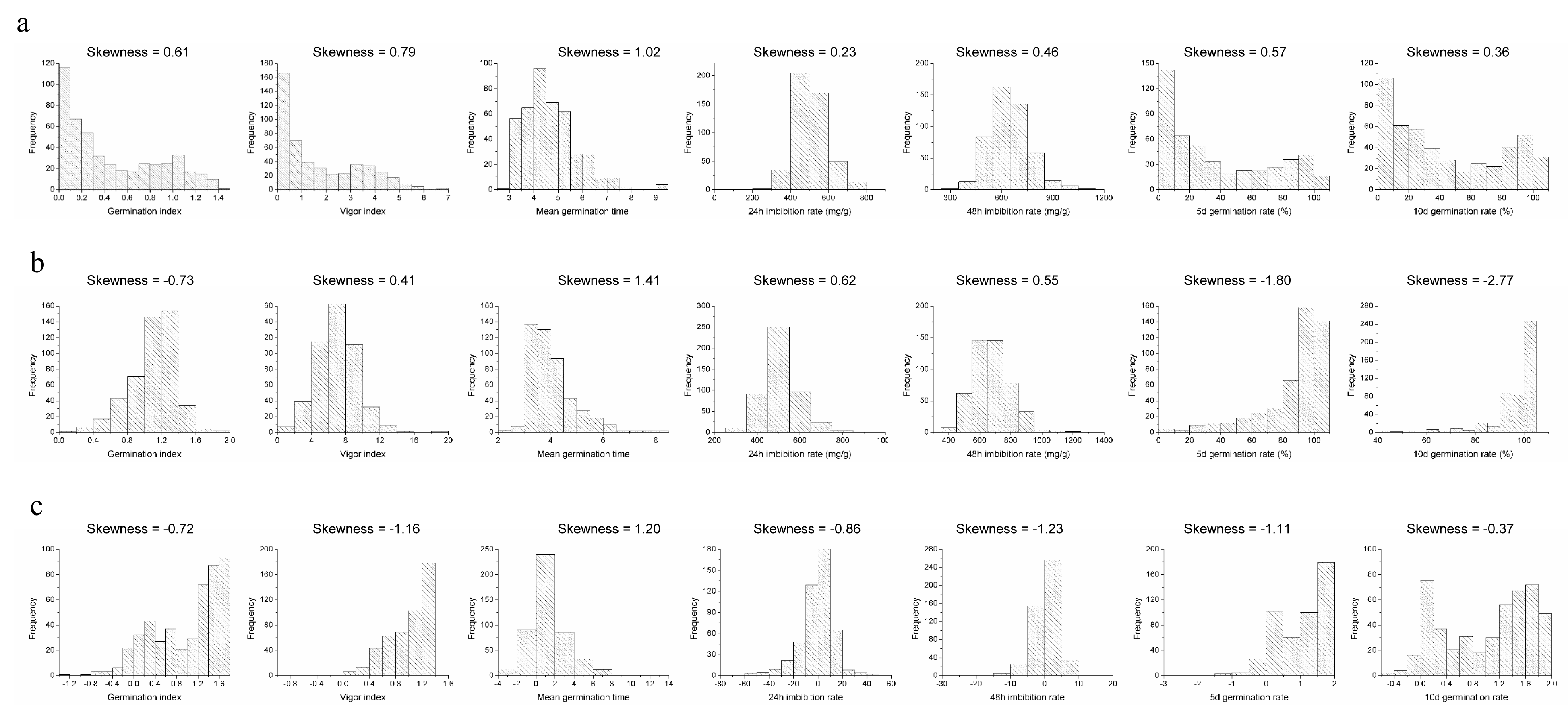

Supplement: Supplementary file 3 — Frequency distributions of germination index, vigor index, mean germination time, imbibition rate, and germination rate for 478 rice accessions under salt-stress and control conditions, and stress susceptibility indices of these traits. Under salt stress (a) and control (b) conditions. Stress susceptibility index (c). (TIFF 1093 kb) [file 12870_2017_1044_MOESM3_ESM.tif]

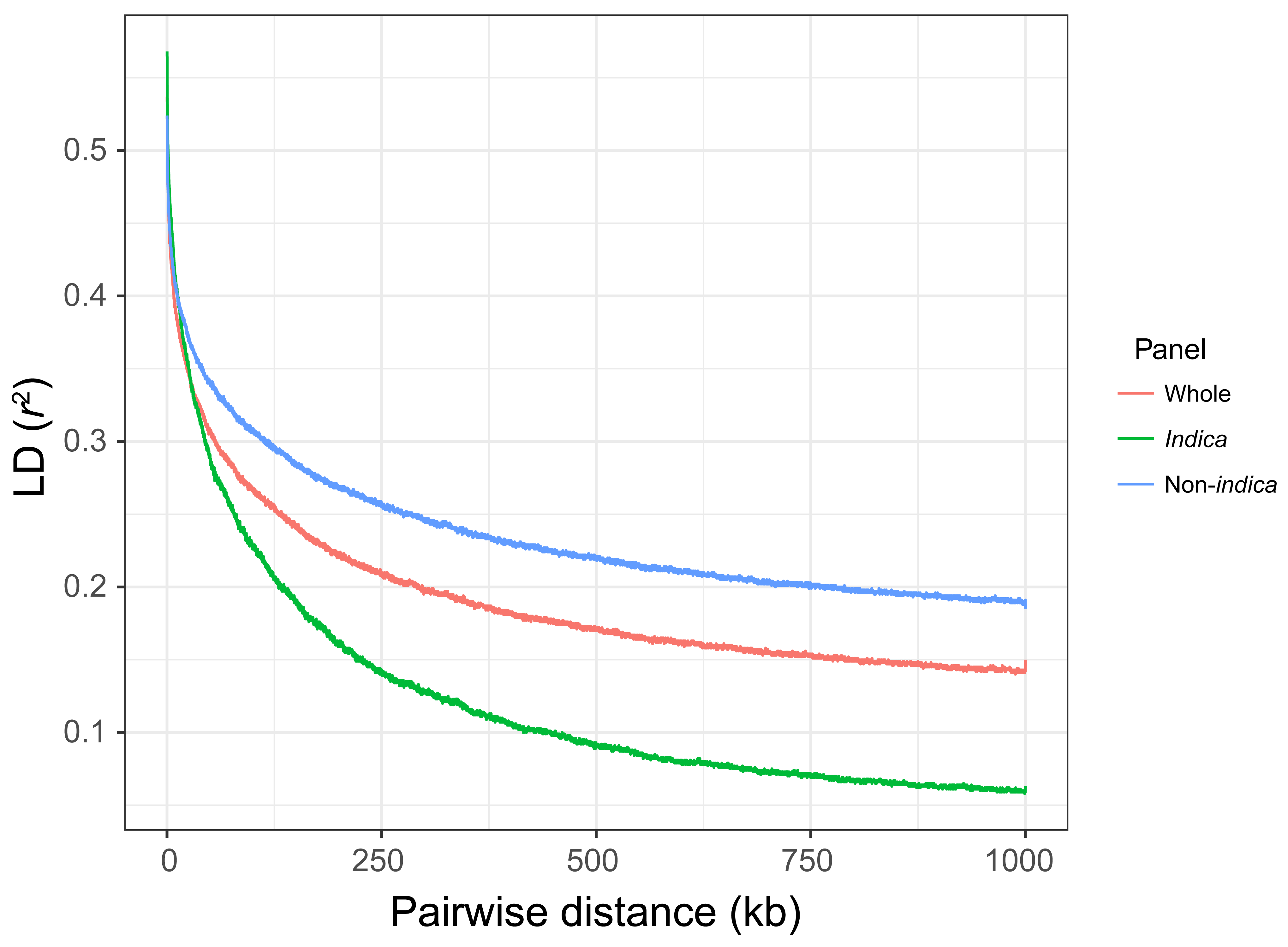

Supplement: Supplementary file 5 — Differences in linkage disequilibrium among panels. (TIFF 422 kb) [file 12870_2017_1044_MOESM5_ESM.tif]
